# Supplementary material for: Weakly supervised learning for multi-organ adenocarcinoma classification in whole slide images
Source: PLoS One. 2022 Nov 23;17(11):e0275378. doi: 10.1371/journal.pone.0275378 (PMC9683606; doi:10.1371/journal.pone.0275378)
Supplement: Attachment — Submitted filename: 20220716_Tsuneki_Response to Reviewers.pdf [file pone.0275378.s001.pdf]

July 16, 2022

RE: Revision of our manuscript (Manuscript ID: PONE-D-22-13432), " Weakly supervised learning for multi-organ adenocarcinoma classification in whole slide images" by Masayuki Tsuneki and Fahdi Kanavati.

Dear Dr. Andrey Bychkov, Academic Editor, PLOS ONE

Thank you very much for your email of July 07, 2022, regarding our manuscript (Manuscript ID: PONE-D-22-13432) together with constructive reviewers' comments.

We are submitting our revised manuscript. Our incorporation of the reviewers' suggestion is stated below.

We believe that we have adequately addressed the concerns raised in the review.

The following is a point-by-point response to the suggestions and concerns raised.

ACADEMIC EDITOR

I wish to reinforce the following comments picked up by the peer reviewers: 1) code availability; 2) issues with figures. When evaluating computational pathology submissions, each reviewer is routinely asked to run the provided code and assess its performance independently, which was not possible in this case.

Response: Regarding code availability, we have further clarified in our response to reviewers. The main point is that we are not proposing a new methodology in this paper, which we explicitly mention in the paper. We are simply doing a clinical application paper, and code is not central to the claim. We do mention that we adapted the code from the tensorflow vision implementation. To avoid any confusion, we have removed this statement. In addition, at this point, there exists various implementations that validate the weakly supervised training with multiple instance learning from a methodology perspective. For instance there is a publicly available implementation from a seminal Nature paper here https://github.com/MSKCC-Computational-Pathology/MIL-nature-medicine-2019

While we appreciate the reviewers being asked to run the code, the bulk of the datasets used in this study are not publicly available without a user agreement. Without the datasets, it would not be possible to ascertain anything about the obtained results. The datasets are large with a combined total of about 9K WSIs, requiring terabytes of storage. Providing the entirety of the dataset would be a challenge. It took weeks to obtain the results from multiple training as it takes a couple of days to train a model on a single GPU.

As far as we are aware, based on the policy of PLOS ONE, it is not a requirement if the code is not central to the manuscript.
https://journals.plos.org/plosone/s/materials-software-and-code-sharing#loc-sharing-code

"In cases where code is central to the manuscript, we may require the code to be made available as a condition of publication. Authors are responsible for ensuring that the code is reusable and well documented"

Regarding the figures, we have explained that it is due to the web-based viewer that adds borders to the tiles which have had a prediction to display the heatmap. This is a visual aid and is proportional to the zoom level, with thicker borders at lower magnifications. We have provided an example video showcasing the viewer.

https://drive.google.com/file/d/167uW8JDOJyfzyRcMP8nxiXx8WuSBmQab/view?usp=sharing

—

Please include the following items when submitting your revised manuscript:

A rebuttal letter that responds to each point raised by the academic editor and reviewer(s). You should upload this letter as a separate file labeled 'Response to Reviewers'.

Response: Done.

A marked-up copy of your manuscript that highlights changes made to the original version. You should upload this as a separate file labeled 'Revised Manuscript with Track Changes'.

Response: We have highlighted in yellow any additions.

An unmarked version of your revised paper without tracked changes. You should upload this as a separate file labeled 'Manuscript'.

Response: OK.

If you would like to make changes to your financial disclosure, please include your updated statement in your cover letter. Guidelines for resubmitting your figure files are available below the reviewer comments at the end of this letter.

If applicable, we recommend that you deposit your laboratory protocols in protocols.io to enhance the reproducibility of your results. Protocols.io assigns your protocol its own identifier (DOI) so that it can be cited independently in the future. For instructions see: https://journals.plos.org/plosone/s/submission-guidelines#loc-laboratory-protocols. Additionally, PLOS ONE offers an option for publishing peer-reviewed Lab Protocol articles, which describe protocols hosted on protocols.io. Read more information on sharing protocols at https://plos.org/protocols?utm_medium=editorial-email&utm_source=authorletters&utm_campaign=protocols.

Journal Requirements:

When submitting your revision, we need you to address these additional requirements.

1. Please ensure that your manuscript meets PLOS ONE's style requirements, including those

for file naming. The PLOS ONE style templates can be found at

https://journals.plos.org/plosone/s/file?id=wjVg/PLOSOne_formatting_sample_main_body.pdf and

https://journals.plos.org/plosone/s/file?id=ba62/PLOSOne_formatting_sample_title_authors_affiliations.pdf

Response: We have used the PLOS ONE latex template.

2. Please provide additional details regarding participant consent. In the Methods section, please ensure that you have specified (1) whether consent was informed and (2) what type you obtained (for instance, written or verbal). If your study included minors, state whether you obtained consent from parents or guardians. If the need for consent was waived by the ethics committee, please include this information

Response: This information was already mentioned in the “Compliance with Ethical Standards” section. For clarity we have separated the part about patient consent into a separate “Patient consent” section.

3. Thank you for stating the following in the Competing Interests section:

"Fahdi Kanavati and Masayuki Tsuneki are employees of Medmain Inc. (Fukuoka, Japan)."

We note that one or more of the authors are employed by a commercial company: Medmain Inc.

Response: We have stated this in the conflict of interest section.

a. Please provide an amended Funding Statement declaring this commercial affiliation, as well as a statement regarding the Role of Funders in your study. If the funding organization did not play a role in the study design, data collection and analysis, decision to publish, or preparation of the manuscript and only provided financial support in the form of authors' salaries and/or research materials, please review your statements relating to the author contributions, and ensure you have specifically and accurately indicated the role(s) that these authors had in your study. You can update author roles in the Author Contributions section of the online submission form.

Response: We have revised the “Funding Statement”.

Please also include the following statement within your amended Funding Statement.

“The funder provided support in the form of salaries for authors [insert relevant initials], but did not have any additional role in the study design, data collection and analysis, decision to publish, or preparation of the manuscript. The specific roles of these authors are articulated in the ‘author contributions’ section.”

Response: We have included this in the Funding statement section of the manuscript.

If your commercial affiliation did play a role in your study, please state and explain this role within your updated Funding Statement.

b. Please also provide an updated Competing Interests Statement declaring this commercial affiliation along with any other relevant declarations relating to employment, consultancy, patents, products in development, or marketed products, etc.

Within your Competing Interests Statement, please confirm that this commercial affiliation does not alter your adherence to all PLOS ONE policies on sharing data and materials by including the following statement: "This does not alter our adherence to PLOS ONE policies on sharing data and materials.” (as detailed online in our guide for authors http://journals.plos.org/plosone/s/competing-interests) . If this adherence statement is not accurate and there are restrictions on sharing of data and/or materials, please state these. Please note that we cannot proceed with consideration of your article until this information has been declared.

Response: We have revised the “Conflict of Interest”.

Please include both an updated Funding Statement and Competing Interests Statement in your cover letter. We will change the online submission form on your behalf.

Response: OK.

4. We note that you have indicated that data from this study are available upon request. PLOS only allows data to be available upon request if there are legal or ethical restrictions on sharing data publicly. For more information on unacceptable data access restrictions, please see http://journals.plos.org/plosone/s/data-availability#loc-unacceptable-data-access-restrictions.

In your revised cover letter, please address the following prompts:

a) If there are ethical or legal restrictions on sharing a de-identified data set, please explain them in detail (e.g., data contain potentially sensitive information, data are owned by a third-party organization, etc.) and who has imposed them (e.g., an ethics committee). Please also provide contact information for a data access committee, ethics committee, or other institutional body to which data requests may be sent.

Response: We have added the sentence “The data contains sensitive information” in the “Availability of data and material” section. I wrote the contact information for the ethics committees in the cover letter (confidential information).

b) If there are no restrictions, please upload the minimal anonymized data set necessary to replicate your study findings as either Supporting Information files or to a stable, public repository and provide us with the relevant URLs, DOIs, or accession numbers. For a list of acceptable repositories, please see http://journals.plos.org/plosone/s/data-availability#loc-recommended-repositories.

We will update your Data Availability statement on your behalf to reflect the information you provide.

5.Your ethics statement should only appear in the Methods section of your manuscript. If your ethics statement is written in any section besides the Methods, please move it to the Methods section and delete it from any other section. Please ensure that your ethics statement is included in your manuscript, as the ethics statement entered into the online submission form will not be published alongside your manuscript.

Response: We have moved this into the methods section.

Reviewers' comments:

Review Comments to the Author

Reviewer #1: The authors use weakly supervised approach for training an algorithm for adenocarcinoma detection.

The study is of potential interest.

There are, however, some important issues:

- Page 2: "(e.g., Hospital-A, B, C, D, and E)"

I would remove word anonymization as this data cannot be anonymized in such way. You can always identify your datasets according to number of slides, staining, cutting peculiarities, etc. What is the meaning of anonymization of hospitals? You probably have to anonymize cases, but not hospitals.

Response: We had a set of five hospitals from which we obtained datasets. By anonymising the dataset, we mean that we do not explicitly mention which dataset originated from a given hospital. So as to avoid associating a particular result to an explicitly named hospital.

- Page 3: What kind of slides did the authors use from TCGA cohort?

Response: We've expanded the sentence to include that were H&E surgical FFPE specimens

As in Table 5 they state to have 246 slides for lung cancer cohort containing only benign tissue, it is probably the slides of fresh frozen sections. This should be clarified. If authors used fresh frozen sections they should provide the clarification for this as they train on FFPE sections and frozen section is a very different type of material.

Response: We have added a mention that the dataset from TCGA contained both FFPE and frozen sections.

- Table 4: Histopathological diagnosis for lymph nodes without metastasis is probably not "Non-neoplastic lesion", just free of tumor.

Response: We have replaced all mention of non-neoplastic lesion with non-neoplastic

- Tumors are extremely heterogenous. How did the pathologists select the cases, how they considered the representativeness. E.g., lung is an organ with a very complex morphology of benign tissue peritumoral. Sometimes you cannot just tell where the tumors starts and where

ends. Fibrosis. Inflammation. Not including enough training data can reduce generalizability of the algorithms.

Response: The criteria for selection was simply whether the WSI contained adenocarcinoma. Otherwise, it was assumed to be non-neoplastic.

- Authors used EfficientNetB1 as the architecture of neural network. It is a relatively small network and not a "state-of-the-art" network for digital pathology tasks as it is known empirically that larger networks (with 20-50 mln of parameters) might perform better. Importantly, authors refer to their other paper (ref. 24) from where they inherit the technical methodology of the current study. And in ref. 24 they used EfficientNetB3. Given this fact, the authors should clarify their selection and support it by experimental data.

Response: We saw no further improvements from using a larger model, which is why we used the B1 model. In previous study, we have also used B1 [1]

[1] Masayuki Tsuneki and Fahdi Kanavati. "Deep learning models for poorly differentiated colorectal adenocarcinoma classification in whole slide images using transfer learning." *Diagnostics* 11.11 (2021): 2074.

- Authors train only batch normalization layers and the classification layer leaving the whole model untrained (authors should state if they used ImageNet weights or not). This seems strange as in most situations for computational pathology you have to retrain the whole model. Although, ImageNet pretrained models can work as feature extractors on pathology tasks, normally one get up to 5% more accuracy when the whole model is retrained (in transfer learning setting).

Response: We have added a mention that it is ImageNet pretrained models. Based on extensive results provided in the partial fine-tuning paper [1], it can be sufficient to only fine-tune the affine weights of the batch normalization layers and the final classification layers. That paper includes comparisons of various fine-tuning/transfer learning approaches on histopathology images.

[1] https://proceedings.mlr.press/v143/kanavati21a.html

- Page 4: "We used k = 8, N = 256, and a batch size of 32". The selection of exactly these hyperparameters should be clarified and supported by experimental evidence.

Response: These parameters had no impact on the final result. They were selected to simply make use of the maximum possible available GPU memory.

- Page 4: Authors start with randomly selecting some patches from training slides (they however do not state, how many patches they use – this should be stated). And train only 1 epoch before switching to hard-mining. By such approach the first selection of random patches can have enormous effects on the result. Authors need to perform some form of bootstrapping or at least present the results from several independent trainings to be able to compare the results among these.

Response: It is the same parameter k that determines the number of selected patches. We are not proposing a new method here and simply using a method that was validated to work in previous studies. In addition, with deep learning with large training and test datasets, it is the general consensus that it is enough to do a single initial split of the data and perform a single

run (See for example Machine Learning Yearning by Andrew Ng), especially when training a single model can take days.

- Page 4: “We used early stopping by tracking the performance of the model on a validation set; this allows stopping the training when no improvement was observed for more than 10 epochs. The model with the lowest validation loss was chosen as the final model.”

This seems to be unclear for me – validation set is practically several reserved biopsies for each tumor entity. The tumor-bearing biopsies contain regions with tumor and with benign tissue. If the authors did not annotate them, the only way to validate their trained models is on the case/slide level. But the authors state they used validation loss as a trigger for stopping training. How exactly did they calculate loss if they have access only to the validation at case/slide level?

Response: This was done on the slide level. The loss is calculated as in any classification problem using binary cross entropy. For the cases in the validation set, we simply applied the model on the entire slide, then took the maximum probability. Now we have a single probability value for the entire slide to compare with the ground truth label. This allows us to compute a loss with the binary cross entropy. We have added a sentence to clarify this.

This should be clarified in all details.

- Code availability:

“To train the classification model in this study we adapted the publicly available 140

TensorFlow training script available at https://github.com/tensorflow/models/ 141

tree/master/official/vision/image_classification.”

By this the authors mean that they will not release their code. If it is so, this should be stated clearly and nor referring the readers to the tensorflow repo! At that, this link is not working. However, my personal opinion is that this is inacceptable for research publication in the field of computational pathology because the others have absolutely no chance to reproduce the results. The methodology of the study is very simple so at least at this point there is, in my opinion, no substantial interference with “commercial background” as authors are affiliated to a company. Anyway, if authors do not provide code, there should be robust clarification for this.

Response: It seems that there have been changes to repo paths, the correct link is https://github.com/tensorflow/models/tree/master/official/vision

At this point, there exists various implementations that validate the weakly supervised training with multiple instance learning. For instance there is a publicly available implementation from a seminal Nature paper here https://github.com/MSKCC-Computational-Pathology/MIL-nature-medicine-2019

We are not proposing a new methodology in this paper. It is simply a clinical application paper, and code is not central to the claim. We do mention that we adapted the code from the tensorflow vision implementation. To avoid any confusion, we have removed this statement.

As far as we are aware, based on the policy of PLOS one, it is not a requirement if the code is

not central to the manuscript.
https://journals.plos.org/plosone/s/materials-software-and-code-sharing#loc-sharing-code

"In cases where code is central to the manuscript, we may require the code to be made available as a condition of publication. Authors are responsible for ensuring that the code is reusable and well documented"

- In Table 6 the authors show the test of the existing (ref. 15) model for stomach adenocarcinoma detection on the test datasets of the current study. On the test dataset from the same domain (stomach) AUC is 0.937. It is totally unclear how authors calculated AUC, whether if they used probability or tumor areas thresholds etc, as they make tests on the case/slide level (non-annotated slides). The same is true for log loss.

Response: We hope the additional paragraph we added to clarify how the slide-level probabilities are computed also answer this question.

Authors show AUC = 0.991 for TCGA breast cancer cohort – however this seems to be totally non-believable as this is an absolutely different pathology domain.

- The same issue (exact method for calculation of AUC and log loss, and other metrics is unclear):

"For each test set (stomach endoscopic biopsy, colon endoscopic biopsy, lung TBLB, breast needle biopsy, radical lymph node dissection, lung TCGA, and breast TCGA), we computed the ROC-AUC, log loss, accuracy, sensitivity, and specificity and summarized the results in Table 7 and 8 and Fig. 2B-D."

- Table 7, Table 8

Again, it is not believable, that you can get AUC 0.999 on such a hard problem like breast cancer training in weakly supervised mode on several hundred biopsies. Probably such results stem from the fact that the authors use only 4 images without tumor for TCGA breast cancer cohort and test at case level. It is a major flaw in design and authors should completely remove TCGA breast dataset from their work.

Response: They do in fact stem from only using 4 images without tumor for the TCGA breast cancer cohort. We have removed this dataset from the results.

- Figures: Authors should clarify what different colors mean (probabilities?)

Response: We have this statement mentioned at the end of each figure caption "the heatmap uses the jet color map where blue indicates low probability and red indicates high probability"

All figures are barely interpretable (the tissue behind color maps cannot be seen)

Response: The exact same image on the right contains the tissue without the color map overlay which allows the reader to see the tissue.

- Authors used datasets from different institutes. At that they do not adopt any normalization strategies. In this case, different number of cases from different institutes can introduce bias and substantially reduce the generalizability. Authors should ideally provide independent

tests using stain normalization strategies. Normally, the results should improve.

Response: We have included the clarification paragraph that we had performed data augmentation on the brightness, contrast, hue and saturation, which would help account for differences in stain. This had previously been mentioned in the earlier paper that described the method in more detail.

- The main achievements of the study (for best models) are presented in Table 8.

Here very low sensitivity and specificity for all tumor entities (TCGA breast should be removed) – actually inacceptable for clinical practice. This reported accuracy corresponds to the inference maps presented in Figures.

Response: We have removed the breast TCGA dataset.

This stems probably at least partially from a very low number of cases (selected for study – histologically very complex and heterogeneous tumor entities). It is known from other publication that for weakly supervised approaches 4000 slides are not enough, the number should be definitively > 10 k, ideally 15k-20k slides. Also methodology is sometimes suboptimal: using very small CNN, not training model (!), only classification layers and batch normalization layers. The authors are encouraged to provide the results for training with stain normalization or style transfer, training of full model as well as training in bootstrapping mode (iterations of random selection of training and test cases).

Response: We again refer the review to a previous publication on the partial transfer learning method https://proceedings.mlr.press/v143/kanavati21a.html for which code is also available (https://github.com/fk128/batchnorm-transferlearning) to reproduce the results of only fine-tuning the affine parameters of batch normalization layers. We have also added a clarification that we did perform data augmentation on the color to make the model less sensitive to stain.

Reviewer #2: The authors present a unique model that is able to classify adenocarcinomas originated from multiple organs in a single model.

Major

1. Breast dataset from TCGA is not appropriate as a test set for cancer detection task, because it is extremely imbalanced (more than 99% are invasive ductal carcinoma).

Response: We have removed the breast TCGA dataset.

2. In the heat map in Figs. 3-7, some tiles have frames while others do not, even in areas where the color does not represent the lowest level. In addition, unnatural white to purple grids are seen in the areas without frames. These raise the suspicion of arbitrary image editing. Please provide original images for the evaluation by editor and reviewers and relevant explanations.

Response: These images were directly extracted from a web-based viewer based on

openseadragon (https://openseadragon.github.io/). The viewer was adapted to display tile overlays of the prediction heatmaps. The predictions were only generated for tissue regions while the rest was automatically assumed to be background. To make tiles easier to visualise at different zoom levels, they had borders added to delineate the tiles to make it easier to view the grid structure of tiles that had prediction. The border lines are proportional to the zoom level. At low magnifications, the border lines are thicker, making them more visible. The more one zooms, the thinner they become, and at maximum magnification, there are no border lines as one can easily see the grid structure of the tiles. This is more of a visual aid. Here is a video showcasing the viewer that shows these borders https://drive.google.com/file/d/167uW8JDOJyfzyRcMP8nxiXx8WuSBmQab/view?usp=sharing

3. In validation and test step, how are the inference results at the patch level aggregated to the WSI level diagnosis?

Response: We have added a paragraph to clarify this.

4. Also, please explain how the thresholds for the inference results were set when calculating sensitivity and specificity.

Response: We used the standard threshold of 0.5.

Minor

1. Why TCGA colon cancer and TCGA gastric cancer datasets were not tested?

Response: We only had the breast and lung TCGA datasets as we had downloaded months prior and reviewed by pathologists. At the time we carried out this study, The TCGA website was not allowing the download of any further images which is why we were unable to obtain other datasets. https://forum.image.sc/t/tcga-slides-not-available-anymore/52532/3

2. What is the “non-neoplastic lesion” in the TCGA dataset? The TCGA dataset is, by definition, dealing with neoplastic lesions. How the datasets chosen for the test set in this study were selected from the TCGA dataset? To clarify the dataset, the "manifest file" used to download the data from the GDC data portal should be made available.

Response: We referred to non-neoplastic lesions as anything that was not neoplastic as reviewed by pathologists. This included things like inflammatory tissue. To further make this clearer we have removed “lesions” so that non-neoplastic simply refers to anything that’s not neoplastic, e.g. non-neoplastic would refer to inflammation and normal.

3. Please explain what cases of metastatic colon cancer of the lungs are in Table 4. Such cases should rarely occur in usual clinical practice.

Response: Here are two representative examples of metastatic colon adenocarcinoma of the lung that we used:

(1) According to the histopathological report, with extensive necrosis, highly atypical columnar epithelial cells proliferate in a fused tubular structure. The patient suffered colon adenocarcinoma and stomach adenocarcinoma as well. Immunohistochemically,

the metastatic lesion in lung was positive for CK20 but negative for CK7. In the same patient, stomach adenocarcinoma was positive for CK7 but negative for CK20; and colon adenocarcinoma was positive for CK20 but negative for CK7. Therefore, final diagnosis for the metastatic lesion was “metastatic colon adenocarcinoma of lung”.

(2) According to the histopathological report, highly atypical columnar epithelial cells take on a fused tubular structure and proliferate nodularly with extensive necrosis. Histologically, this is a finding of colorectal cancer metastasis. The same patient suffered colon adenocarcinoma in his/her clinical history.

4. There should be cases where there are multiple WSIs in a single case. How these cases were handled?

Response: We only used a single WSI from a given case.

5. On which dataset is the deep learning model pretrained?

Response: Tables 1 and 2 show which datasets the model was trained on.

6. In “code availability” section, please provide author-generated code.

Response: At this point, there exists various implementations that validate the weakly supervised training with multiple instance learning. For instance there is a publicly available implementation from a seminal Nature paper here https://github.com/MSKCC-Computational-Pathology/MIL-nature-medicine-2019

We are not proposing a new methodology in this paper. It is simply a clinical application paper, and code is not central to the claim. We do mention that we adapted the code from the tensorflow vision implementation. To avoid any confusion, we have removed this statement.

As far as we are aware, based on the policy of PLOS one, it is not a requirement if the code is not central to the manuscript.
https://journals.plos.org/plosone/s/materials-software-and-code-sharing#loc-sharing-code

“In cases where code is central to the manuscript, we may require the code to be made available as a condition of publication. Authors are responsible for ensuring that the code is reusable and well documented”

Reviewer #3: The manuscript presents research with important impact and objective, e.g. to provide an auxiliary tool to diagnostic pathology and improve global adenocarcinoma primary screening. I found the approach to be original in aiming to identify adenocarcinoma with considerable accuracy in several primary sites and metastatic lymph nodes, hence matching the diagnostic practice and protocol in multidisciplinary centers with high throughput. Using weakly supervised learning is a strong point in this research foe a list of reasons: Less time in achieving the results without using pathologists annotation efforts, it still remains comprehensive and explainable for the target user , and most importantly achieving very good performance. Below are a list of minor improvements I would suggest :

- The writing used is straight to the point and gets the message across very well, however I would suggest to replace one sentence in the Introduction : Adenocarcinoma is the major cancer arise in these 7

organs, with -Adenocarcinoma is the most common type of cancer affecting these 7 organs.

Response: Done.

-Throughout the manuscript is consistently used " histopathological cancer classification" which I understand is linked to the technical realm of computational pathology, but from a practicing pathologist perspective cancer classification goes beyond identification of adenocarcinoma and can be miss interpreted from the reader. I would suggest to replace with " tissue classification and adenocarcinoma detection".

Response: Done.

- I appreciated the Method and materials section as it is detailed, well explained and figures show the right data at a glance, however here I was not clear about the validation set size. I found it to be small, but I am not an image analysis expert, and it would be great to add a sentence about the size of validation set criteria if possible.

Response: Tables 1 and 2 show the size of the validation sets in parentheses. The test set represents the hold out sets and they're detailed in Tables 3,4, and 5.

- It was not entirely clear why the adenomas were pointed out in the class of non-adenocarcinoma for GI (stomach and colon). I would suggest to explain in a sentence or two the rational for that. e.g. high risk feature , or most common differential etc.

Response: We have included it because it's the most common differential diagnosis as well as to ensure that the model predicts correctly the difference between adenoma and adenocarcinoma due to potential similarity in some features. We have added a sentence to highlight this.

- I would suggest to be more broad when listing the limitations of the study, in addition to the false positives and false negatives. Considering the aim of being impactful in primary screening, the use of only one type of scanner is another limitation to be mentioned for example.

Response: We have added a sentence about the limitations due to the scanner and false positives/negatives.

—

Sincerely yours,

Masayuki Tsuneki

Board Director & VP of Medical Research

Medmain Research, Medmain Inc.

2-4-5-104, Akasaka, Chuo-ku, Fukuoka, 810-0042 Japan

Phone: +81-92-707-1977 (office)

E-mail: tsuneki@medmain.com
