# Supplementary material for: Weakly supervised learning for multi-organ adenocarcinoma classification in whole slide images
Source: PLoS One. 2022 Nov 23;17(11):e0275378. doi: 10.1371/journal.pone.0275378 (PMC9683606; doi:10.1371/journal.pone.0275378)
Supplement: Attachment — Submitted filename: 20220821_Tsuneki_Response to Reviewers.pdf [file pone.0275378.s002.pdf]

August 21, 2022

RE: Revision of our manuscript (Manuscript ID: PONE-D-22-13432R1), " Weakly supervised learning for multi-organ adenocarcinoma classification in whole slide images" by Masayuki Tsuneki and Fahdi Kanavati.

Dear Dr. Andrey Bychkov, Academic Editor, PLOS ONE

Thank you very much for your email of August 17, 2022, regarding our manuscript (Manuscript ID: PONE-D-22-13432R1) together with reviewers' comments.

We are submitting our revised manuscript. Our incorporation of the reviewers' suggestion is stated below.

We believe that we have adequately addressed the concerns raised in the review.

---

6. Review Comments to the Author

Reviewer #1: Authors addressed most of the points and discussed those they are not able to address.

The manuscript can be accepted in the actual form.

Response: Thank you.

Reviewer #2: Authors removed TCGA breast data this round, however, the data remain in manuscript. Authors should fix them all before submission.

Response: Thank you so much. We found breast TCGA data, so we've removed it. The remaining mentions of breast refer to the needle biopsy set that's not from TCGA.

As the reviewer comment before, colon cancer rarely metastasizes to lung lymph node. (Of cause, metastasis to lung is very common in contrast.) These two examples the authors had shown also seem the cases of metastasis to lung (rather than lymph node metastasis). Please discuss again with your consultant pathologists.

Response: We've discussed it again with the pathologists. Though it might be rare, those two examples do correspond to colon cancer metastasis to lung lymph node.

It is still unclear what is the "non-neoplastic" slides from TCGA. Authors need to open the lists of cases for both adenocarcinoma and non-neoplastic subgroup, ideally with the inference result for each case. Otherwise, there is no sense in using publicly available data. (Authors did not response the original comment to open manifest file of GDC data portal the authors used for this analysis.)

Response: We had downloaded the data a while back and imported a copy into our cloud-based viewer. Unfortunately we didn't keep a copy of the open manifest file. In line with that, we have removed the results as well from the lung TCGA dataset. We have amended Figure 2 by excluding the lung TCGA set.

The result/figure authors claim are all based on an algorithm that authors have developed yourselves. (If there is nothing new in the methodology, then just use the existing code that is publicly available.) There is no doubt that codes are very important in this regard.

PLOS expect all researchers to share author-generated code for reproducibility and reuse. The reviewer hopes the authors to share your code.

Response: We do believe that we are still in line with PLOS policy as the code is not central to the manuscript given that we are not proposing a new methodology and there already exists some publicly available code that implements a similar methodology [1].

https://journals.plos.org/plosone/s/materials-software-and-code-sharing#loc-sharing-code

"In cases where code is central to the manuscript, we may require the code to be made available as a condition of publication. Authors are responsible for ensuring that the code is reusable and well documented"

[1] https://github.com/MSKCC-Computational-Pathology/MIL-nature-medicine-2019

—

Sincerely yours,

Masayuki Tsuneki

Board Director & VP of Medical Research

Medmain Research, Medmain Inc.

2-4-5-104, Akasaka, Chuo-ku, Fukuoka, 810-0042 Japan

Phone: +81-92-707-1977 (office)

E-mail: tsuneki@medmain.com
