# Supplementary material for: Weakly supervised learning for multi-organ adenocarcinoma classification in whole slide images
Source: PLoS One. 2022 Nov 23;17(11):e0275378. doi: 10.1371/journal.pone.0275378 (PMC9683606; doi:10.1371/journal.pone.0275378)
Supplement: Attachment — Submitted filename: 20220914_Tsuneki_Response to Reviewers.pdf [file pone.0275378.s003.pdf]

September 14, 2022

RE: Revision of our manuscript (Manuscript ID: PONE-D-22-13432R2), " Weakly supervised learning for multi-organ adenocarcinoma classification in whole slide images" by Masayuki Tsuneki and Fahdi Kanavati.

Dear Dr. Andrey Bychkov, Academic Editor, PLOS ONE

Thank you very much for your email of September 13, 2022, regarding our manuscript (Manuscript ID: PONE-D-22-13432R2) together with the reviewer's comments.

We are submitting our revised manuscript. Our incorporation of the reviewer's suggestion is stated below.

We believe that we have adequately addressed the concerns raised in the review.

---

Journal Requirements:

Please review your reference list to ensure that it is complete and correct. If you have cited papers that have been retracted, please include the rationale for doing so in the manuscript text, or remove these references and replace them with relevant current references. Any changes to the reference list should be mentioned in the rebuttal letter that accompanies your revised manuscript. If you need to cite a retracted article, indicate the article's retracted status in the References list and also include a citation and full reference for the retraction notice.

**Response:** It is complete and correct.

Reviewers' comments:

6. Review Comments to the Author

Reviewer #2:

- Authors need to clearly state the major limitation in the Discussion that the models were not validated in independent cohort(s) from different institutions.

**Response:** We have added this as the last sentence in the discussion.

- Fix in in the Abstract that the models were evaluated in five (not seven) independent test sets.

**Response:** Fixed. Thank you so much.

—

Sincerely yours,

Masayuki Tsuneki

Board Director & VP of Medical Research

Medmain Research, Medmain Inc.

2-4-5-104, Akasaka, Chuo-ku, Fukuoka, 810-0042 Japan

Phone: +81-92-707-1977 (office)

E-mail: tsuneki@medmain.com
